# Serum lipid profile among sporadic and familial forms of Parkinson’s disease

**DOI:** 10.1038/s41531-021-00206-6

**Published:** 2021-07-16

**Authors:** Daniel Macías-García, María Teresa Periñán, Laura Muñoz-Delgado, María Valle Jimenez-Jaraba, Miguel Ángel Labrador-Espinosa, Silvia Jesús, Dolores Buiza-Rueda, Carlota Méndez-Del Barrio, Astrid Adarmes-Gómez, Pilar Gómez-Garre, Pablo Mir

**Affiliations:** 1 Unidad de Trastornos del Movimiento Servicio de Neurología y Neurofisiología Clínica, Instituto de Biomedicina de Sevilla Hospital Universitario Virgen del Rocío/CSIC/Universidad de Sevilla, Seville, Spain; 2 Centro de Investigación Biomédica en Red sobre Enfermedades Neurodegenerativas (CIBERNED), Madrid, Spain; 3 Departamento de Medicina Facultad de Medicina, Universidad de Sevilla, Seville, Spain

**Keywords:** Parkinson's disease, Parkinson's disease

## Abstract

Brain cholesterol metabolism has been described as altered in Parkinson’s disease (PD) patients. Serum lipid levels have been widely studied in PD with controversial results among different populations and age groups. The present study is aimed at determining if the serum lipid profile could be influenced by the genetic background of PD patients. We included 403 PD patients (342 sporadic PD patients, 30 *GBA-*associated PD patients, and 31 *LRRK2-*associated PD patients) and 654 healthy controls (HCs). Total cholesterol, HDL, LDL, and triglycerides were measured in peripheral blood. Analysis of covariance adjusting for sex and age (ANCOVA) and post hoc tests were applied to determine the differences within lipid profiles among the groups. Multivariate ANCOVA revealed significant differences among the groups within cholesterol and LDL levels. *GBA-*associated PD patients had significantly lower levels of total cholesterol and LDL compared to *LRRK2-*associated PD patients and HCs. The different serum cholesterol levels in *GBA-*associated PD might be related to diverse pathogenic mechanisms. Our results support the hypothesis of lipid metabolism disruption as one of the main PD pathogenic mechanisms in patients with *GBA-*associated PD. Further studies would be necessary to explore their clinical implications.

## Introduction

Parkinson’s disease (PD) is the most common neurodegenerative disorder after Alzheimer’s disease^1^. The pathognomonic hallmark of PD is the degeneration of dopaminergic neurons of the substantia nigra and, consequently, the striatal dopamine depletion. Although it has been studied for years, PD etiology still remains uncertain. Several factors, environmental as well as genetic, have been elucidated as key factors in PD pathogenesis^2^. These factors lead to the degeneration of dopaminergic neurons through different metabolic pathways (oxidative stress, mitochondrial dysfunction, iron accumulation, endosomal–lysosomal functioning, among others).

Among environmental factors, lipids seem to play a role in the neurodegeneration of PD^3–5^. Brain cholesterol metabolism has been described as altered in PD patients and plasma 24-OH-cholesterol has been considered as a possible biomarker for PD^6,7^. In addition, studies with cultured neurons and animal models suggested that certain lipids and their metabolites promote an increase in oxidative stress and alpha-synuclein (α-syn) aggregation in dopaminergic cells^8,9^.

Different studies have shown controversial results regarding serum lipid levels in PD^10–15^. A large prospective cohort study described an increased risk of PD with hypercholesterolemia among subjects aged under 55 years^10^. However, other studies showed lower levels of cholesterol, low-density lipoprotein (LDL), apolipoprotein-B, and triglycerides in PD patients, suggesting a protective factor of lipids in the PD course^11,13^. Moreover, two prospective studies described a decrease in PD risk between subjects with hypercholesterolemia^15,16^. A possible influence on the disease course and the cognitive state in PD was proposed for serum cholesterol and LDL levels^17,18^. Recently, in a meta-analysis of genome-wide association studies of PD, it was demonstrated that lipids and lipoproteins were involved in the pathogenic mechanisms of the disease, such as oxidative stress response or lysosomal functioning^19^. Interestingly, they found shared genetic etiology between lipid rafts total cholesterol and triglycerides and PD. These studies suggest a causal relationship between cholesterol metabolism and PD.

Several genes have been associated with the familial forms of PD. The identification of these genes and their mechanisms provides an insight into PD etiopathology^20^. Leucine-rich repeat kinase 2 gene (*LRRK2*) and glucocerebrosidase (*GBA*) gene are two of the most common causes of familial PD; however, mutations in those genes are found in different proportions among the PD population worldwide (from 0.4 to 20%)^21,22^.

The low penetrance of the most common *LRRK2* mutation (p.G2019S) suggested that there should be other factors that modulate the dopaminergic degeneration^23^. A higher peripheral inflammation and altered lipid storage capacity were suggested as underlying mechanisms in *LRRK2* pathogenesis^24,25^. Moreover, *LRRK2* knock-out animal models have shown changes in their serum cholesterol levels, whereas higher triglyceride levels have been described in *LRRK2* carriers^26,27^. On the other hand, heterozygous *GBA* mutations cause a loss of function of glucocerebrosidase (GCase) in PD patients, showing these patients with *GBA* variants a specific phenotype with higher cognitive decline and autonomic dysfunction than sporadic PD^28^. A link between the loss of function of GCase and lysosomal cholesterol accumulation was described in fibroblasts of PD patients with the *GBA* p.N370S mutation^29^. Moreover, patients with Gaucher type I disease, carriers of homozygous *GBA* mutations, showed reduced serum levels of cholesterol, LDL, and high-density lipoprotein (HDL)^30^. Besides, studies in animal models suggested that a perturbation in glycosphingolipid levels might induce neuroinflammation and accelerate the PD pathogenesis in vulnerable dopaminergic neurons^5,31^.

Given the role of lipids in PD pathogenesis and the possible influence of *LRRK2* and *GBA* in that relationship, the present study is aimed at determining whether there are differences in the serum lipid profile between sporadic PD (sPD) and the main monogenic causes of familial PD (*GBA-*associated PD, *GBA*-PD and *LRRK2-*associated PD, *LRRK2-*PD). We hypothesized that levels of the serum lipids could be influenced by the genetic background of PD patients.

## Results

### Case–control study

The demographic characteristics and the serum lipid profile of the whole PD cohort and healthy controls (HCs) are summarized in Table 1. PD patients were older than HCs (65.07 ± 11.83 vs 58.77 ± 16.01, *p* <0.005), with a slight predominance of males in both groups (52.14 and 58.31% of males, respectively). Regarding the serum lipid profile among both groups, serum levels of total cholesterol (TC), LDL, and triglycerides were lower in PD patients than HCs. Differences in TC and triglycerides levels between groups were statistically significant after adjusting for age and sex. There were no significant differences in HDL levels between PD patients and HCs.

**Table 1:** Demographic characteristic and lipid profile of healthy controls and Parkinson’s disease patients.

	Healthy controls (*n* = 420)	PD (*n* = 300)	*P* value
Sex (% males)	52.14 %	58.31 %	0.09
Age (y), mean ± SD	58.77 ± 16.01	65.07 ± 11.83	<0.005
TC (mg/dl), mean ± SD	202.15 ± 38.11	194.84 ± 41.35	0.029*
HDL (mg/dl), mean ± SD	58.37 ± 17.25	58.96 ± 16.61	0.69
LDL (mg/dl), mean ± SD	127.49 ± 30.44	124.9 ± 38.73	0.49
TG (mg/dl), mean ± SD	115.59 ± 70.14	103.03 ± 57.68	0.006*

*TC* total cholesterol, *HDL* high-density lipoprotein, *LDL* low-density lipoprotein, *TG* triglycerides

*PD* Parkinson’s disease patients, *SD* standard deviation.

*<0.05 after adjusting by sex and age.

### Parkinson’s disease cohorts study

The demographic and clinical characteristics of the different PD cohorts (sPD, *GBA*-PD, and *LRRK2-*PD), as well as HCs are shown in Table 2. The familial PD groups (*GBA*-PD and *LRRK2*-PD) were younger than sPD and showed a lower age of PD onset. *GBA*-PD had a predominance of males (73.91%), whereas *LRRK2-*PD showed a higher proportion of females (62.98%). Although these PD groups had a longer disease duration than sPD, there were no differences between groups either in the severity of the disease or in the treatment. There were no significant differences in arterial hypertension, diabetes, or hyperlipidemia among the groups (Table 2).

**Table 2:** Demographic and clinical data of healthy controls and Parkinson’s disease cohorts.

	HCs (*n* = 420)	sPD (*n* = 250)	*GBA*-PD (*n* = 23)	*LRRK2*-PD (*n* = 27)	*P* value
Sex (% males)	52.14%	58.40%	73.91%	37.02	0.03**
Age (y), Mean ± SD	58.77 ± 16.09	65.92 ± 11.77	58.83 ± 11.8	63.41 ± 11.79	<0.005**
Arterial hypertension, *n* (%)	139 (33%)	104 (42%)	8 (35%)	8 (30%)	0.17
Diabetes, *n* (%)	52 (12%)	45 (18%)	1 (4%)	2 (7%)	0.08
Hyperlipidemia, *n* (%)	57 (14%)	47 (19%)	2 (9%)	6 (22%)	0.19
Age of onset (y), mean ± SD	–	56.60 ± 11.95	48.39 ± 8.87	51.65 ± 12.86	0.007*
Disease duration (y), mean ± SD	–	8.9 ± 6.23	10.67 ± 7.69	11.81 ± 7.06	<0.05*
Hoehn & Yahr, mean ± SD	–	2.26 ± 1.22	2.5 ± 0.96	1.9 ± 2.4	0.16
LEDD, mean ± SD	–	764.74 ± 470.14	694.25 ± 408.76	797.46 ± 579.46	0.86

*HCs* healthy controls, *LEDD* levodopa equivalent daily dose, *sPD* sporadic Parkinson’s disease patients, *GBA*-*PD* *GBA*-associated Parkinson’s disease patients, *LRRK2-PD* *LRRK2-*associated Parkinson’s disease patients, *SD* standard deviation.

*p<0.05 **p<0.005.

When we compared the serum lipid profile among PD cohorts and HCs, we found statistically significant differences in TC levels [*F* (3,663) = 4.99, *P* < 0.005] and LDL [*F* (3,502) = 3.85, *P* <0.05] after adjusting for age and sex (Table 3). *GBA*-PD showed the lowest levels of TC and LDL (178.22 ± 34.47 and 105.78 ± 29.85 mg/dl, respectively), while *LRRK2-*PD patients had the highest levels of both TC and LDL (213.73 ± 30.01 and 141.09 ± 30.48 mg/dl, respectively). TC and LDL levels of sPD were lower than HCs and *LRRK2-*PD but higher than the levels of *GBA*-PD patients. No differences were found between the groups in HDL levels. Regarding the triglycerides, HCs showed higher serum triglycerides levels than sPD, *GBA*-PD, and *LRRK2-*PD. However, these differences in triglycerides were not statistically significant after adjusting by sex and age [*F* (3,658) = 2.06, *P* = 0.10].

**Table 3:** Serum lipid profile in healthy controls and Parkinson’s disease cohorts.

	HCs	sPD	*GBA*-PD	*LRRK2*-PD	*F value*	*P* value
TC (mg/dl), mean ± SD	202.15 ± 38.11	195.76 ± 42.68	178.22 ± 34.47	213.73 ± 30.01	4.99	0.002**
HDL (mg/dl), mean ± SD	58.37 ± 17.25	60.46 ± 17.77	56.11 ± 14.25	56.22 ± 12.32	1.03	0.38
LDL (mg/dl), mean ± SD	127.49 ± 30.44	125.75 ± 40.65	105.78 ± 29.85	141.09 ± 30.48	3.85	0.01*
TG (mg/dl), mean ± SD	115.59 ± 70.14	102.03 ± 59.63	105.39 ± 42.84	110.19 ± 63.46	2.06	0.10

*TC* total cholesterol, *HDL* high-density lipoprotein, *LDL* low-density lipoprotein, *TG* triglycerides, *HCs* healthy controls, *sPD* sporadic Parkinson’s disease patients, *GBA-PD* *GBA*-associated Parkinson’s disease patients, *LRRK2-PD* *LRRK2-*associated Parkinson’s disease patients, *SD* standard deviation.

Analysis of covariance (ANCOVA), adjusting by sex and age, was applied. **p<0.005 *p<0.05.

### Multiple comparisons

**Fig. 1:**
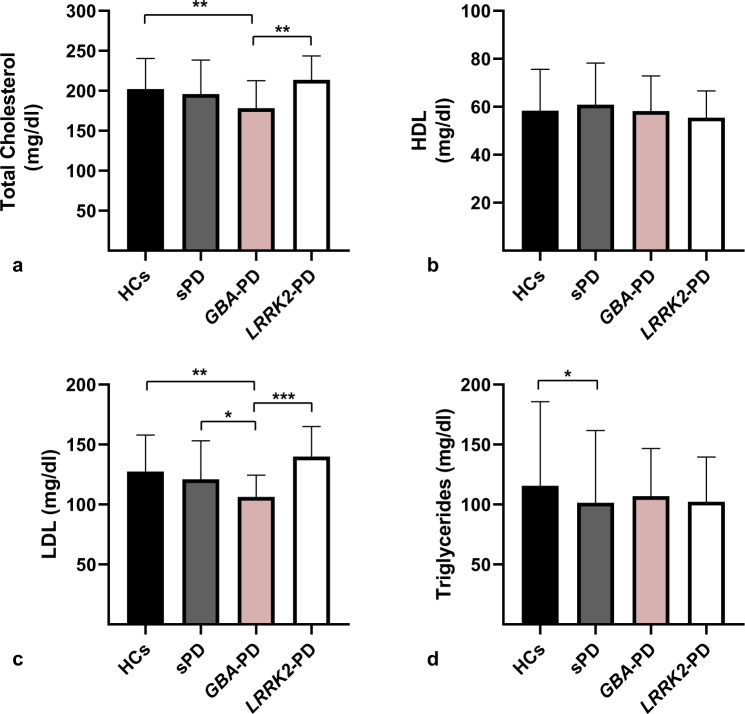
Serum lipid profile among Parkinson’s disease cohorts and healthy controls. Multiple comparisons of total cholesterol (**a**), HDL (**b**), LDL (**c**), and triglycerides (**d**) levels among healthy controls (HCs), sporadic Parkinson’s disease patients (sPD), *GBA*-associated Parkinson’s disease patients (*GBA*-PD), and *LRRK2*-associated Parkinson’s disease patients (*LRRK2*-PD). Numbers shown as means ± SD with one-way ANCOVA with post hoc testing for multiple comparisons as appropriate. ***p<0.005, **p<0.05, *p < 0.1.

We accomplished multiple comparisons to determine the differences in the lipid profile between groups (Figs. 1a–d). The complete results of Levene’s test and post hoc tests are described in Supplementary Data 1 (Supplementary Tables 1–6). Post hoc analysis showed that TC and LDL levels were lower in *GBA*-PD compared with *LRRK2-*PD (*p* <0.05) (Figs. 1a, 1c). However, no differences were found in HDL or triglyceride levels.

When we compared the familial PD groups and sPD, we observed that LDL levels of *GBA*-PD were lower than in the sPD group. This difference remained marginally significant (*p* = 0.07). *GBA*-PD TC levels were also lower than sPD; however, no statistically significant differences were found. Regarding the *LRRK2-*PD group, we observed higher levels of both TC and LDL levels in *LRRK2-*PD patients compared to sPD. Nevertheless, these differences did not achieve statistical significance (*p* = 0.12 and *p* = 0.16, respectively). No differences were found between the familial PD groups and sPD in HDL or triglycerides.

Finally, post hoc analysis showed statistically significant differences in TC and LDL levels between *GBA*-PD and HCs. Regarding triglycerides, sPD patients showed lower levels than HCs (Fig. 1d), although these differences remained marginally significant (*p* = 0.059).

## Discussion

The role of serum lipids in PD pathogenesis remains unclear with different results in several studies. This study specifically analyzed serum cholesterol profiles comparing familial and sporadic forms of PD. Our data indicate that *GBA*-PD patients had lower serum cholesterol and LDL levels than *LRRK2*-PD patients and HCs. Previous studies have shown that women had higher HDL and lower total cholesterol levels than men^32,33^. In this line, our findings are particularly noticeable ever since *GBA-*PD was the group with the highest male proportion and *LRRK2*-PD had a female predominance. Sex influence appears not to play the main role in the serum lipid levels of these patients, suggesting a possible link between specific PD pathogenesis and lipid metabolism. Moreover, a different serum lipid profile was observed in *GBA*-PD compared with sPD even though differences observed were marginally significant.

Our results in the case–control study showed lower levels of cholesterol and triglycerides in patients with PD compared to HCs, as previously described^11–14^. However, after PD subtyping, our largest PD group (sPD) showed lower triglycerides but no significant differences in cholesterol levels compared with HCs (Figs. 1a, 1d). Rozani et al. recently demonstrated a decrease in PD risk in middle-aged men and elderly women with hypercholesterolemia in the largest statin-free prospective study of serum lipids and PD^15^. Klemann et al. found a shared genetic risk between lipid/lipoprotein levels and PD^19^. In the same line, a Mendelian randomization study showed an association between higher levels of serum lipids (TC, LDL, and triglycerides) and a lower future risk of PD^16^. These studies suggest a causal relationship between lipid profile and PD. On the other hand, a large prospective cohort study showed an increased risk of PD with hypercholesterolemia among subjects aged under 55 years^10^. These controversial results could be explained by the heterogeneity within PD patients included in each study. Our results in the different PD cohorts showed that patients with *GBA*-PD have a different serum lipid profile, therefore supporting the idea that lipid metabolism could be influenced by PD pathogenesis. Our findings could explain the inconsistencies previously found within validation studies with larger PD populations, since lipid profiles could be influenced by the genetic background of PD patients. In this line, some authors proposed a different approach based on a better PD subtyping to achieve the identification of novel biomarkers and disease-modifying treatments in PD^34,35^.

Recently, the idea of PD as a protein-induced lipidopathy has been proposed, rather than as a proteinopathy^36^. This fresh approach to the Lewy pathology (LP) is based on new insights into α-syn inclusions near the lipid rafts of cellular membranes^37,38^. Lipid rafts are cholesterol- and sphingolipids-enriched microdomains of cellular membranes that coordinate bioactivity of membrane constituents and protein-lipid interactions^39^. The altered composition of lipids rafts in frontal cortex neurons of PD patients has been described, compared with healthy controls^40^. Also, the main proteins involved in monogenic forms of PD, such as LRRK2 or parkin, have been associated with lipid rafts and its dysfunction in PD pathology^41,42^. Nevertheless, our findings in *LRRK2*-PD patients do not allow us to draw conclusions about the role of lipid metabolism disruption in the specific pathophysiology of patients with *LRRK2* mutations.

Although a link between *GBA* variants and PD is well established in different populations, the relationship between GCase and α-syn pathology remains uncertain^43–47^. A decreased GCase activity is supposed to promote LP and modulate neuronal susceptibility to α-syn seeding by disruption in lysosomal maturation^48–50^. An in vitro study of PD patient fibroblasts with the *GBA* p.N370S mutation has evidenced a link between the loss of function in GCase and the lysosomal cholesterol accumulation with the appearance of multilamellar bodies (MLBs)^29,51^. It has been proposed that the cholesterol needed for the formation of MLBs originates from two different routes: (1) endogenous cholesterol synthesis in the endoplasmic reticulum and (2) LDL-containing cholesterol that binds to the LDL-receptor on the cell membrane^48^. Interestingly, the *GBA* p.N370S mutation was the most prevalent of our pathogenic variants included in the *GBA*-PD group. This fact provides support to the idea of disrupted cholesterol metabolism in *GBA*-PD, which alters the autophagy-lysosome function and produces MLBs, being a possible explanation of the specific lipid profile observed in our patients^50^. Besides, some authors have stated the need of viewing PD as a systemic disorder where global cellular processes (pathogenic mechanisms) occur in a range of cellular subtypes, and not only in those cell types that display the characteristic neuropathology (i.e., dopaminergic neurons)^52^. To date, it remains unknown whether cholesterol accumulation is the first step in *GBA*-PD pathogenesis, or if it occurs secondarily to other lipids accumulations (i.e., glycosphingolipids)^51^. MLBs contain both primarily undegraded phospholipids and cholesterol. In this line, Guedes et al. reported decreased phospholipids levels (phosphatidic acid, phosphatidylethanolamine, among others) in the serum of *GBA* mutation carriers^53^. However, in the same study, increased levels of ceramides were reported in those patients. Finally, the lower TC and LDL levels in our *GBA-*PD patients are in line with the previous description of decreased cholesterol levels in Gaucher disease type I patients^30^. As we mentioned above, our work highlights the role of cholesterol metabolism and its link with *GBA-*PD pathology.

It is reasonable to consider whether lipid metabolism disruption (as a pathogenic mechanism of *GBA*-PD) could influence the specific phenotype of patients with *GBA*-PD^28,43,44^. However, answering this question is beyond the scope of this study and our study design does not allow us to evidence it. Longitudinal studies might be necessary to explore the influence of lipid profile in the *GBA*-PD course.

Regarding other aspects of the lipid profile in our study, neither HDL nor triglyceride levels differed between the familial PD groups and sPD. Differences in triglycerides were observed in our case–control analysis with decreased levels in the whole PD cohort compared to HCs. Nevertheless, when we classified in familial and sporadic PD groups, only sPD patients showed a decrease in triglyceride levels, which remained marginally significant compared with HCs. Our results might suggest that decreased triglyceride levels might be linked to sporadic forms of PD rather than genetic forms. These findings are contrary to those observed by Thaler et al^27^. These authors accomplished an interesting study wherein they analyzed the possible influence of the metabolic syndrome in *GBA-* and *LRRK2-*associated PD patients compared with sPD. Although no relationship was observed between metabolic syndrome and disease course among PD groups, they described elevated triglyceride levels in *LRRK2* carriers. Interestingly, they also measured HDL levels as part of the metabolic syndrome, and no differences were found between familial and sporadic forms of PD. This former result is in line with our study, suggesting a lack of relationship between PD and HDL.

The strength of our conclusions is tempered by certain limitations. Firstly, our study is retrospective and we cannot elude that some other variables out of our control might influence the study, so our results require to be interpreted cautiously. Although most of the main factors that can directly influence the serum lipid levels were taken into account with selected inclusion/exclusion criteria (i.e., ethnic influence and lipid-modifying therapies), others, such as the body mass index or the type of diet of the study participants, were not considered. However, no relationship between dietary cholesterol intake and PD risk was previously found in a meta-analysis, either in prospective or case-control studies^54^. Second, we studied neither prodromal PD patients nor *GBA*/*LRRK2-*mutated asymptomatic carriers. Thus, we were not able to study the effect of these mutations in the lipid profile before motor symptoms of PD manifest. Further studies with those prodromal patients might be required to confirm if the genetic background of PD patients influences their serum cholesterol levels. Finally, our familial PD cohorts have a relatively small sample size compared with sPD or HCs, limiting the statistical power for further analysis.

In conclusion, our results show that *GBA-*associated PD patients have different serum lipid profiles, and support the hypothesis of lipid metabolism disruption as one of the main pathogenic mechanisms in *GBA-*associated PD. However, further investigations would be necessary to confirm these findings and to study their possible clinical implications.

## Methods

### Participants

We included 403 PD patients who were classified into three subgroups: 342 sporadic PD patients (sPD), 30 *GBA*-associated PD patients (*GBA*-PD), and 31 *LRRK2-*associated PD patients (*LRRK2-*PD). A control group with 654 HCs was also included. All subjects considered for the study were Caucasian to avoid ethnic influences in the lipid profile. PD patients were recruited from the Movement Disorder Clinic at Hospital Universitario Virgen del Rocio in Seville, Spain. PD was diagnosed following the Movement Disorder Society Clinical Diagnostic Criteria^55^. HCs were recruited from the same geographical area and they were not considered for the study if they had any neurodegenerative disorder, a family history of PD, or a variant in *GBA* or *LRRK2* genes. sPD patients were not considered for the study if they had any variants in the PD-related genes. The *LRRK2-*PD group contained 28 *LRRK2* p.G2019S PD patients (90.3%) and 3 *LRRK2* p.R1441G PD patients (9.7%). The list of *GBA* pathogenic variants considered for the inclusion of patients in the *GBA*-PD group is shown in Table 4.

**Table 4:** List of GBA pathogenic variants considered for the inclusion of patients in the *GBA*-associated Parkinson’s disease group.

Allele	cDNA	Protein	Exon	*n*
D409H	c.1342 G/C	p.Asp448His	10	2
L444P	c.1448 T/C	p.Leu483Pro	11	4
N370S	c.1223 A/G	p.Asn409Ser	10	12
S310G	c.928 A/G	p.Ser310Gly	8	3
L29fs	c.84dupG	p.Leu29Alafs*18	3	1
R535H	c.1604G > A	p.Arg535His	12	1
V457D	c.1487 T/A	p.Val496Asp	11	2
G195W	c.700 G > T	p.Gly234Trp	7	2
F213I	c.754 T > A	p.Phe252Ile	7	2
R262C	c.901 C > T	p.Arg301Cys	8	1

*cDNA* complementary DNA

**Fig. 2:**
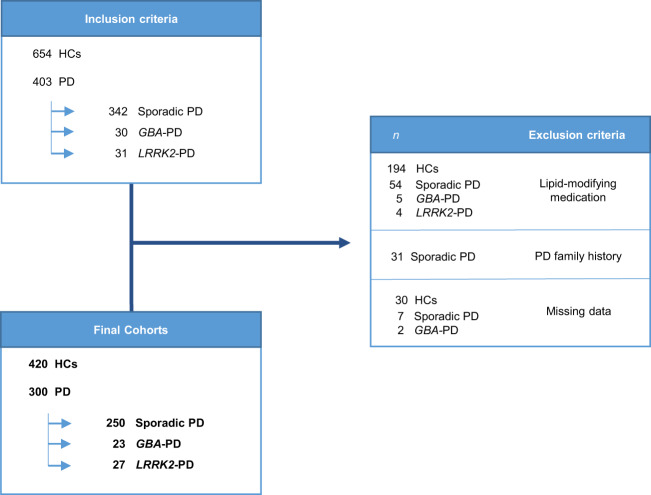
Study flowchart with selection criteria and final cohorts. HCs healthy controls, *GBA*-PD *GBA-*associated Parkinson’s disease patients, *LRRK2*-PD *LRRK2*-associated Parkinson’s disease patients, *PD* Parkinson’s disease.

Exclusion criteria for all individuals included were receiving treatment with lipid-modifying therapy (i.e., statins, ezetimibe, antivirals, azathioprine), and having a first-degree PD family history exclusively for sPD patients. Figure 2 shows the study flowchart, exclusion criteria, and resultant cohorts.

We obtained consent from the local ethics committee of our hospital in accordance with the Declaration of Helsinki, and written informed consent from all the participants in the study. All subjects underwent a clinical assessment at our center and the demographical data were retrospectively obtained by consulting their previous medical records. Serum TC, HDL, LDL, and triglycerides were determined in peripheral blood and analyzed in the Central Laboratory of our center.

### Genetics

Genomic DNA was isolated from peripheral blood samples by standard or automated methods (DNA Isolation Kit for Mammalian Blood, Roche, Maxwell 16 System, Promega Corporation, Madison, WI, USA; MagNA Pure LC, Roche Diagnostics, Indianapolis) in compliance with established protocols. The mutational screening of *GBA* and *LRRK2* genes was previously done using a combination of high-resolution melting (HRM) and direct DNA resequencing as we describe below^22,43^.

Here we describe the procedure followed to do the *GBA* screening. Twenty-five nanograms of genomic DNA was used for each polymerase chain reaction (PCR). To prevent amplification of the neighboring pseudogene, *GBA* was first amplified in four large fragments that only and specifically amplified the functional gene but not the nearby pseudogene. PCR primer couples were designed based on the known genomic sequence (NG_009783.1). The list with the primers used in our study for *GBA* screening is shown in Supplementary Data 2 (Supplementary Table 7). For the mutational screening, we studied isoform 1 of the *GBA gene* (NM_001005741. 2), which contains 12 exons, including a noncoding exon 1. The mutational screening of all exons and intron-exon boundaries was then performed, using a combination of HRM analysis and direct DNA resequencing. HRM reactions were performed on a LightCycler480 (LC480) instrument, and HRM curve acquisition and analysis were performed using LC480 software version 1.3 (Roche Applied Science, Indianapolis, IN, USA). Samples showing abnormal melting profiles, including those with variants, were sequenced on both strands using the BigDye terminator cycle sequencing kit (Applied Biosystems, Foster City, CA, USA) and resolved on an ABI3500 genetic analyzer (Applied Biosystems). We have adopted the conventional nomenclature, which refers to the processed protein and excludes the 39-residue signal peptide.

The *LRRK2* screening was done following the procedure described below. The mutational screening of all exons and intron–exon boundaries was performed using a HRM analysis and/or targeted resequencing. The gene regions which encode functional domains of LRRK2 begin within exon 22 (amino acid 984), so, in this study, we used HRM analysis to screen for variations from that exon until exon 51. HRM reactions were performed on a LC480 instrument, and HRM curve acquisition and analysis were performed using LC480 software version 1.3 (Roche Applied Science). All samples showing abnormal melting profiles were sequenced by Sanger sequencing. The list with the primers used in our study for *LRRK2* screening by HRM is shown in Supplementary Data 2 (Supplementary Table 8). Targeted resequencing was performed using a customized Haloplex Target Enrichment Panel (including *LRRK2*), which was designed using Agilent’s online Sure Design tool, following the manufacturer’s protocol (Agilent Technologies, Inc., Santa Clara, CA, USA). Samples were sequenced employing the Illumina NextSeq platform (Illumina Inc., San Diego, CA, USA). Filtered variants predicted as pathogenic were validated by Sanger sequencing. To determine the origin of each patient with R1441G mutation, haplotype analysis was performed by testing microsatellite markers and single-nucleotide polymorphisms spanning the gene region.

### Statistical analysis

Group comparisons of categorical variables were performed using chi-square and Fisher’s tests. Firstly, we compared the serum lipid profile (TC, LDL, HDL, and triglycerides) between the total PD group and HCs using multivariate linear regression adjusting for sex and age. The homogeneity of variance was studied with Levene’s test. Second, we compared the serum lipids levels between the different PD cohorts (sPD, *GBA*-PD, and *LRRK2-*PD) and HCs using the analysis of covariance (ANCOVA), considering age and sex as covariates. Then, post hoc analysis for multiple comparisons between groups was applied. All statistical analyses were performed using IBM SPSS software (26 for Windows; IBM, Armonk, NY) and GraphPad Prism 8 software (GraphPad Software, Inc.). A *p* value <0.05 was considered statistically significant.

### Reporting Summary

Further information on research design is available in the Nature Research Reporting Summary linked to this article.

## Supplementary information

Supplementary Information

Reporting Summary

## Data Availability

The data that support the findings of this study are available from the corresponding author upon reasonable request.

## References

[1] Hirtz D (2007). How common are the ‘common’ neurologic disorders?. Neurology.

[2] Obeso JA (2017). Past, present, and future of Parkinson’s disease: a special essay on the 200th Anniversary of the Shaking Palsy. Mov. Disord..

[3] Jin U, Park SJ, Park SM (2019). Cholesterol metabolism in the brain and its association with Parkinson’s disease. Exp. Neurobiol..

[4] Xicoy H, Wieringa B, Martens GJM (2019). The role of lipids in Parkinson’s disease. Cells.

[5] Hallett PJ, Engelender S, Isacson O (2019). Lipid and immune abnormalities causing age-dependent neurodegeneration and Parkinson’s disease. J. Neuroinflammation.

[6] Orth M, Bellosta S (2012). Cholesterol: its regulation and role in central nervous system disorders. Cholesterol.

[7] Huang X (2019). Brain cholesterol metabolism and Parkinson’s disease. Mov. Disord..

[8] Paul R, Choudhury A, Borah A (2015). Cholesterol – a putative endogenous contributor towards Parkinson’s disease. Neurochem. Int..

[9] Halliday GM (2005). Synuclein redistributes to neuromelanin lipid in the substantia nigra early in Parkinson’s disease. Brain.

[10] Hu G, Antikainen R, Jousilahti P, Kivipelto M, Tuomilehto J (2008). Total cholesterol and the risk of Parkinson disease. Neurology.

[11] Guo X (2015). The serum lipid profile of Parkinson’s disease patients: a study from China. Int. J. Neurosci..

[12] Wei Q (2013). Reduced serum levels of triglyceride, very low density lipoprotein cholesterol and polipoprotein B in Parkinson’s disease patients. PLoS ONE.

[13] de Lau LML, Koudstaal PJ, Hofman A, Breteler MMB (2006). Serum cholesterol levels and the risk of Parkinson’s disease. Am. J. Epidemiol..

[14] Scigliano G (2006). Reduced risk factors for vascular disorders in Parkinson disease patients: a case-control study. Stroke.

[15] Rozani V (2018). Higher serum cholesterol and decreased Parkinson’s disease risk: a statin-free cohort study. Mov. Disord..

[16] Fang F (2019). Lipids, apolipoproteins, and the risk of Parkinson disease. Circ. Res..

[17] Huang X (2011). Serum cholesterol and the progression of Parkinson’s disease: results from DATATOP. PLoS ONE.

[18] Sterling NW (2016). Higher plasma LDL-cholesterol is associated with preserved executive and fine motor functions in Parkinson’s disease. Aging Dis..

[19] Klemann CJHM (2017). Integrated molecular landscape of Parkinson’s disease. npj Park. Dis..

[20] Kim C, Alcalay R (2017). Genetic forms of Parkinson’s disease. Semin. Neurol..

[21] Trinh J (2018). Genotype-phenotype relations for the Parkinson’s disease genes SNCA, LRRK2, VPS35: MDSGene systematic review. Mov. Disord..

[22] Gao L (2009). Prevalence and clinical features of LRRK2 mutations in patients with Parkinson’s disease in southern Spain. Eur. J. Neurol..

[23] Lee AJ (2017). Penetrance estimate of LRRK2 p.G2019S mutation in individuals of non-Ashkenazi Jewish ancestry. Mov. Disord..

[24] Cabezudo D, Baekelandt V, Lobbestael E (2020). Multiple-hit hypothesis in Parkinson’s disease: LRRK2 and inflammation. Front. Neurosci.

[25] Yu M (2018). LRRK2 mediated Rab8a phosphorylation promotes lipid storage. Lipids Health Dis..

[26] Baptista MAS (2013). Loss of leucine-rich repeat kinase 2 (LRRK2) in rats leads to progressive abnormal phenotypes in peripheral organs. PLoS ONE.

[27] Thaler A (2020). Metabolic syndrome does not influence the phenotype of LRRK2 and GBA related Parkinson’s disease. Sci. Rep..

[28] Avenali M (2019). Evolution of prodromal parkinsonian features in a cohort of GBA mutation-positive individuals: a 6-year longitudinal study. J. Neurol. Neurosurg. Psychiatry.

[29] García-Sanz P (2017). N370S-GBA1 mutation causes lysosomal cholesterol accumulation in Parkinson’s disease. Mov. Disord..

[30] Ginsberg H (1984). Reduced plasma concentrations of total, low density lipoprotein and high density lipoprotein cholesterol in patients with Gaucher type I disease. Clin. Genet..

[31] Hallett PJ (2018). Glycosphingolipid levels and glucocerebrosidase activity are altered in normal aging of the mouse brain. Neurobiol. Aging.

[32] Freedman DS (2004). Sex and age differences in lipoprotein subclasses measured by nuclear magnetic resonance spectroscopy: the Framingham study. Clin. Chem..

[33] Klingel SL (2017). Sex differences in blood HDL-c, the total cholesterol/HDL-c ratio, and palmitoleic acid are not associated with variants in common candidate genes. Lipids.

[34] Espay AJ, Lang AE (2018). Parkinson diseases in the 2020s and beyond: replacing clinico-pathologic convergence with systems biology divergence. J. Park. Dis..

[35] Lawton M (2018). Developing and validating Parkinson’s disease subtypes and their motor and cognitive progression. J. Neurol. Neurosurg. Psychiatry.

[36] Fanning S, Selkoe D, Dettmer U (2020). Parkinson’s disease: proteinopathy or lipidopathy?. npj Park. Dis..

[37] Shahmoradian SH (2019). Lewy pathology in Parkinson’s disease consists of crowded organelles and lipid membranes. Nat. Neurosci..

[38] Sulzer D, Edwards RH (2019). The physiological role of α‐synuclein and its relationship to Parkinson’s Disease. J. Neurochem..

[39] Lingwood D, Simons K (2010). Lipid rafts as a membrane-organizing principle. Science.

[40] Fabelo N (2011). Severe alterations in lipid composition of frontal cortex lipid rafts from Parkinson’s disease and incidental Parkinson’s disease. Mol. Med..

[41] Hatano T (2007). Leucine-rich repeat kinase 2 associates with lipid rafts. Hum. Mol. Genet..

[42] Cha S-H (2015). Loss of parkin promotes lipid rafts-dependent endocytosis through accumulating caveolin-1: implications for Parkinson’s disease. Mol. Neurodegener..

[43] Jesús S (2016). GBA variants influence motor and non-motor features of Parkinson’s disease. PLoS ONE.

[44] Alcalay RN (2015). Glucocerebrosidase activity in Parkinson’s disease with and without GBA mutations. Brain.

[45] De Melo Amaral CE (2019). GBA mutations p.n370s and p.l444p are associated with Parkinson’s disease in patients from northern Brazil. Arq. Neuropsiquiatr..

[46] Nuytemans K (2020). Novel variants in LRRK2 and GBA identified in latino Parkinson disease cohort enriched for Caribbean origin. Front. Neurol..

[47] Gegg ME (2015). No evidence for substrate accumulation in Parkinson brains with GBA mutations. Mov. Disord..

[48] García-Sanz, P., Aerts, M. F. G. J. & Moratalla, R. The role of cholesterol in α-synuclein and Lewy body pathology in GBA1 Parkinson’s disease. *Mov. Disord*. **36**,1–17 (2020). 10.1002/mds.28396 PMC8247417 33219714

[49] Henderson MX (2020). Glucocerebrosidase activity modulates neuronal susceptibility to pathological α-synuclein insult. Neuron.

[50] García-Sanz P, Moratalla R (2018). The importance of cholesterol in Parkinson’s disease. Mov. Disord..

[51] García-Sanz P, Orgaz L, Fuentes JM, Vicario C, Moratalla R (2018). Cholesterol and multilamellar bodies: lysosomal dysfunction in GBA -Parkinson disease. Autophagy.

[52] Reynolds RH (2019). Moving beyond neurons: the role of cell type-specific gene regulation in Parkinson’s disease heritability. npj Park. Dis..

[53] Guedes LC (2017). Serum lipid alterations in GBA-associated Parkinson’s disease. Park. Relat. Disord..

[54] Wang A, Lin Y, Wu Y, Zhang D (2015). Macronutrients intake and risk of Parkinson’s disease: a meta-analysis. Geriatr. Gerontol. Int..

[55] Postuma RB (2015). MDS clinical diagnostic criteria for Parkinson’s disease. Mov. Disord..

