# Supplementary material for: Serum lipid profile among sporadic and familial forms of Parkinson’s disease
Source: NPJ Parkinsons Dis. 2021 Jul 16;7:59. doi: 10.1038/s41531-021-00206-6 (PMC8285472; doi:10.1038/s41531-021-00206-6)
Supplement: Supplementary file 1 — Supplementary Information [file 41531_2021_206_MOESM1_ESM.pdf]

SUPPLEMENTARY DATA

Supplementary Data 1. Statistical Analysis Methods

We accomplished a Levene's test to study the homogeneity of variances of serum lipid variables (Total Cholesterol, HDL, LDL and triglycerides) in our population. Depending on the results of the test, we chose the proper *post hoc* comparisons test and, if it was necessary, we added a Welch´s test to the analysis of covariance (ANCOVA) already done. If there was homogeneity of variances (Levene's test *p* value > 0.05), we applied Tukey test for multiple comparisons between groups. Instead, if homogeneity of variances should not be accepted (Levene's test *p* value < 0.05), we applied Games-Howell test for multiple comparisons.

Supplementary Table 1. Test for Equality of Variances (Levene's) in serum lipid variables

	**F**	**df1**	**df2**	**p**
Total cholesterol	0.850	3	665	0.193
HDL	1.130	3	529	0.336
LDL	5.091	3	519	0.002*
Triglycerides	2.375	3	660	0.069

* p < 0.05

We applied Welch's test to compare mean levels of serum lipids variables between groups when homogeneity of variances was not accepted (LDL).

Supplementary Table 2. Welch's Test applied to compare mean levels of serum lipids variables between groups

	**F**	**df1**	**df2**	***p***
Total Cholesterol	5.902	3	63.039	0.001*
HDL	1.029	3	52.265	0.388
LDL	4.620	3	50.232	0.006*
Triglycerides	2.199	3	64.098	0.097

* p < 0.05

Supplementary Table 3. Post Hoc Comparisons - Total Cholesterol

		Mean Difference	95% CI for Mean Difference		SE	t	p tukey
			Lower	Upper			
HCs	sPD	6.397	-2.09	14.89	3.296	1.940	0.212
	GBA-PD	23.935	2.22	45.65	8.430	2.838	0.024**
	LRRK2-PD	-11.578	-32.07	8.91	7.957	-1.226	0.465
sPD	*GBA*-PD	17.538	-4.64	39.72	8.613	2.035	0.176
	LRRK2-PD	-17.975	-38.97	3.02	8.150	-1.993	0.123
GBA-PD	*LRRK2*-PD	-35.513	-64.50	-6.53	11.253	-3.004	0.009**

Note. P-value and confidence intervals adjusted for comparing a family of 4 estimates (confidence intervals corrected using the tukey method). HCs: healthy controls. sPD: sporadic Parkinson's disease. *GBA*-PD: *GBA*-associated Parkinson's disease patients. *LRRK2*-PD: *LRRK2*-associated Parkinson's disease patients. *** p< 0.005 ****** p< 0.05 *p<0.1

Supplementary Table 4. Post Hoc Comparisons - HDL

		Mean Difference	95% CI for Mean Difference		SE	t	p tukey
			Lower	Upper			
HCs	sPD	-2.083	-6.36	2.20	1.661	-1.741	0.303
	GBA-PD	-2.261	-8.43	12.95	4.147	-0.175	0.948
	LRRK2-PD	2.155	-7.37	11.68	3.695	1.211	0.937
sPD	*GBA*-PD	4.344	-6.65	15.34	4.267	0.514	0.739
	LRRK2-PD	4.238	-5.63	14.11	3.829	1.932	0.686
GBA-PD	*LRRK2*-PD	-0.106	-14.01	13.80	5.395	0.962	1.000

Note. P-value and confidence intervals adjusted for comparing a family of 4 estimates (confidence intervals corrected using the tukey method). HCs: healthy controls. sPD: sporadic Parkinson's disease. *GBA*-PD: *GBA*-associated Parkinson's disease patients. *LRRK2*-PD: *LRRK2*-associated Parkinson's disease patients. *** p< 0.005 ****** p< 0.05 *p<0.1

Supplementary Table 5. Games-Howell Post Hoc Comparisons - LDL

**Comparison**		**Mean Difference**	**95% CI for Mean Difference**		**SE**	**t**	**p $_{G-H}$**
			**Lower**	**Upper**			
HCs	sPD	1.733	-7.98	11.45	3.754	0.462	0.967
	GBA-PD	21.708	1.35	42.06	7.241	2.998	0.034**
	LRRK2-PD	-13.601	-31.69	4.49	6.581	-1.62	0.191
sPD	*GBA*-PD	19.975	-1.43	41.40	7.790	2.564	0.074*
	LRRK2-PD	-15.334	-34.687	4.018	7.180	-1.73	0.162
GBA-PD	*LRRK2*-PD	-35.309	-60.812	-9.806	9.482	-3.39	0.003***

HCs: healthy controls. sPD: sporadic Parkinson's disease. *GBA*-PD: *GBA*-associated Parkinson's disease patients. *LRRK2*-PD: *LRRK2*-associated Parkinson's disease patients. *** p< 0.005 ****** p< 0.05 *p<0.1

Supplementary Table 6. Post Hoc Comparisons - Triglycerides

		**Mean Difference**	**95% CI for Mean Difference**		**SE**	**t**	**p** $_{tukey}$
			**Lower**	**Upper**			
HCs	sPD	13.563	0.393	29.596	5.533	2.451	0.059*
	GBA-PD	10.199	-24.382	48.258	14.060	0.725	0.875
	LRRK2-PD	5.398	-29.152	39.418	13.311	0.407	0.980
sPD	*GBA*-PD	-3.364	-40.254	34.140	14.377	-0.234	0.995
	LRRK2-PD	-8.165	-44.989	25.267	13.607	-0.600	0.925
GBA-PD	*LRRK2*-PD	-4.801	-55.400	41.791	18.768	-0.256	0.994

Note. P-value and confidence intervals adjusted for comparing a family of 4 estimates (confidence intervals corrected using the tukey method). HCs: healthy controls. sPD: sporadic Parkinson's disease. *GBA*-PD: *GBA*-associated Parkinson's disease patients. *LRRK2*-PD: *LRRK2*-associated Parkinson's disease patients.
***p<0.005 **p< 0.05 *p<0.1

Supplementary Data 2.

Supplementary Table 7. Complete list of primers used in GBA screening

Primer	Sequences
Exon 1A F	5’-GGAAGGTTAGGAATCCTCTGAGC
Exon 1A R	5’-GAAAAGCAGCCCTGGGGAGT
Exon 1B f	5’-GCCTCTGCATGAGTGACCGT
Exon 1B r	5’-TTAAGTGCGAACGCAGGGAG
Exon 2 F	5’-CCGGAATTACTTGCAGGGCT
Exon 2 R	5’-CCTGGATTCAAAGAGAGTCTGTCA
Exon 3 F2	5’-GTGGGCCTTGTCCTAATGAA
Exon 3 R2	5’-ACCACCTGCTTACTGGAAGG
Exon 4 F	5’-GCAGATGTGTCCATTCTCCA
Exon 4 R	5’-CACTGACACCATTTACCTCTAGGA
Exon 5 F	5’-TTCCCGCTGGGTACTGATAC
Exon 5 R	5’-CGAAAAGTTTCAATGGCTCT
Exon 6 F	5’-CAGGAGCCCAAGTTCCCTTT
Exon 6 R	5’-CTACAGTTTCTCAACCCCCAGA
Exon 7 F	5’-GTGGAGGCTAATGGCTGAAC
Exon 7 R3	5’-ACAGATCAGCATGGCTAAAT
Exon 8 F	5’-CACCCAGCTGGTCTGGTC
Exon 8 R	5’-GCTCTAAGTTTGGGAGCCAGT
Exon 9 F	5’-CTGTGTGCAAGGTCCAGGAT
Exon 9 R	5’-AGGTCTGAGGTCTGCTTTGC
Exon 10 F2	5’-CACAGCTGCCTCTCCCACAT
Exon 10 R4	5’-GTCACTTCCTGCCTCCATG
Exon 11 F	5’-GTCCGTGGGTGGGTGACT
Exon 11 R	5’-ACGCTGTCTTCAGCCCACT
Exon 12 F2	5’-TGGCAGGATCACACTCAG
Exon 12 R2	5’-TGCTGTGCCCTCTTTAGTCA

Supplementary Table 8. Complete list of primers used in LRRK2 screening

**Primer**	**Sequences**
Exon 20 F2	AATTGTTGATTTCTAAGTTGCTGGT
Exon 20 R2	TGGGTCCTATTGTTCAATGTCAGT
Exon 21 F	GATTTTACAAAGGGAATGGACTGTG
Exon 21 R	GTCAGCAGCAAAACACAACATAATC
Exon 22 F	TGTCCTCTTCTCCAATAAATGACAG
Exon 22 R	TGGAGGAAATTCAACCAAACAC
Exon 23 F	GCTAGGAGGTGCTCACTAAACTTT
Exon 23R	AAGCTCTTCAAATGTCTTGAAAGTT
Exon 24 F	GCTAGACTTAAGTTCCTCAGATGG
Exon 24 R	TCAGCATATTTAGGCAACCC
Exon 25 F	AATGAGTCCTCTTTGATGCTGTTC
Exon 25 R	AAGGGTCCATATATGACTCATCTTG
Exon 26 F	CACTATTGGTAGCTGTTCTTATTTTTG
Exon 26R	AAGGTTCTGTTCCAGCTAATGTG
Exon 27 F	ACCCTGGGGAAAATTATTTGTG
Exon 27 R	GAAGCTTCTAGTTTCATGAAATTGG
Exon 28 F	CTTCCTTCCCACCAACAGG
Exon 28 R	TGTCCATCAAAGTCACAGAGAG
Exon 29 F	CAAATACTAGGTTTCTTCAACAGCG
Exon 29 R	CATACAGTCTACCAGGTTTCTGGAT
Exon 30 F	GGATTCTTGCCTGTCGTTTG
Exon 30 R	CTCGGAAAGTTTCCCAATTCAA
Exon 31 F	AGCAGGCCCAGTTTGAAAG
Exon 31 R	GACATTTCTAGGCAGTTGAGAATC
Exon 32 F	TTAGCACTGAATTTGCCAACC
Exon 32 R	CCGTATGGATATTCTCTCAACTTTG
Exon 33 F	AAAGCCCCTTGATATTTGTTC
Exon 33 R	ATGCTTTGACCATAACCCCC
Exon 34 F	TCTTTCTGACTACTTTCACTGAGCA
Exon 34 R	TTCTTTACCTGCTTGGAACCAG
Exon 35 F	AGGTTGGGTGTTTTGTGAGG
Exon 35 R	ATGCCATCTCCCTAATTTCTC
Exon 36 F	GGAAGCAGTTAATAATTAATGGCTC
Exon 36 R	AAACATTCAAATTGTTTCCTTACC
Exon 37 F	CTTTGCGACAGTATGAGGTTTAGA
Exon 37 R	TGAAGGATCACTTAAAAGCATTTGT
Exon 38 F	TTCACATCAAAACCACAAATTTATG

Exon 38 R	GCAAAACAAATTCCAGATCTGTAAT
Exon 39 F	TCAATGAAACAAGTAGGTCAGGTTT
Exon 39 R	CATTATTTTGCATTTTCACAACTGT
Exon 40 F	CCTGTTGATGCACTTTAAAGAAGG
Exon 40 R	TGACCTACTTACCTCAAACCATCAG
Exon 41 F	TTTTGATGCTTGACATAGTGGAC
Exon 41 R	CACATCTGAGGTCAGTGGTTATC
Exon 42 F	TATGAGCCCTGATGTTGGTC
Exon 42 R	CAATTAAATAAAAATGAAGCTGCTG
Exon 43 F	TTTTCTTTGCAATGTCTGGACC
Exon 43 R	GGTGAAAATTATAGGAGGTTTGCC
Exon 44 F	TTCAAGGGAAATGAGTTAACTCG
Exon 44 R	TGAAAGATTTAAAGAGCATTTGGA
Exon 45 F	TTTTCTTTTCTTAACAGGAAGTTGC
Exon 45 R	TGCTGATGCAATAACAATTATGC
Exon 46 F	AAAGTGGAGGAGAACATTAAGGC
Exon 46 R	AATCCCATAAGAGGGGTGTG
Exon 47 F	TTTGAAAGCACAGATTTTATGGAG
Exon 47 R	AAGATCTTCCTTATGAATTATCAACAG
Exon 48 F	ATATTCAATTCAGAATGGTTAGGGA
Exon 48 R	CTGTGACACATGAAGTGCAAAGAT
Exon 49 F	CTTTATGGTTCTAGGGAGGTAATGG
Exon 49 R	GACACTGGAAAGTTCAGAGAAAATG
Exon 50 F	TTCAGTTCCAAGGTATTTGTGTC
Exon 50 R	TGTTACCATCATTCACATCATTG
Exon 51 F	TAAAAATACATGAGCCAAACTGAAA
Exon 51 R	TTCATACACACGAGCTATTTCAAAA
