# Supplementary material for: Serum lipid profile among sporadic and familial forms of Parkinson’s disease
Source: NPJ Parkinsons Dis. 2021 Jul 16;7:59. doi: 10.1038/s41531-021-00206-6 (PMC8285472; doi:10.1038/s41531-021-00206-6)
Supplement: Supplementary file 2 — Reporting Summary [file 41531_2021_206_MOESM2_ESM.pdf]

nature research

Corresponding author(s): DBPR / NPJPARKD-00664

Last updated by author(s): Apr 18, 2021

Reporting Summary

Nature Research wishes to improve the reproducibility of the work that we publish. This form provides structure for consistency and transparency in reporting. For further information on Nature Research policies, see our Editorial Policies and the Editorial Policy Checklist.

Statistics

For all statistical analyses, confirm that the following items are present in the figure legend, table legend, main text, or Methods section.

n/a	Confirmed	
☐	☒	The exact sample size (n) for each experimental group/condition, given as a discrete number and unit of measurement
☐	☒	A statement on whether measurements were taken from distinct samples or whether the same sample was measured repeatedly
☒	☐	The statistical test(s) used AND whether they are one- or two-sided<br>*Only common tests should be described solely by name; describe more complex techniques in the Methods section.*
☐	☒	A description of all covariates tested
☐	☒	A description of any assumptions or corrections, such as tests of normality and adjustment for multiple comparisons
☐	☒	A full description of the statistical parameters including central tendency (e.g. means) or other basic estimates (e.g. regression coefficient) AND variation (e.g. standard deviation) or associated estimates of uncertainty (e.g. confidence intervals)
☐	☒	For null hypothesis testing, the test statistic (e.g. F, t, r) with confidence intervals, effect sizes, degrees of freedom and P value noted<br>*Give P values as exact values whenever suitable.*
☒	☐	For Bayesian analysis, information on the choice of priors and Markov chain Monte Carlo settings
☒	☐	For hierarchical and complex designs, identification of the appropriate level for tests and full reporting of outcomes
☒	☐	Estimates of effect sizes (e.g. Cohen's d, Pearson's r), indicating how they were calculated

Our web collection on statistics for biologists contains articles on many of the points above.

Software and code

Policy information about availability of computer code

Data collection	No software for data collection was used.
Data analysis	All statistical analyses were performed using IBM SPSS software (26 for Windows; IBM, Armonk, NY) and GraphPad Prism 8 software (GraphPad Software, Inc.).

For manuscripts utilizing custom algorithms or software that are central to the research but not yet described in published literature, software must be made available to editors and reviewers. We strongly encourage code deposition in a community repository (e.g. GitHub). See the Nature Research guidelines for submitting code & software for further information.

Data

Policy information about availability of data

All manuscripts must include a data availability statement. This statement should provide the following information, where applicable:

- Accession codes, unique identifiers, or web links for publicly available datasets
- A list of figures that have associated raw data
- A description of any restrictions on data availability

The data that support the findings of this study are available from the corresponding author upon reasonable request.

Field-specific reporting

Please select the one below that is the best fit for your research. If you are not sure, read the appropriate sections before making your selection.

☒ Life sciences ☐ Behavioural & social sciences ☐ Ecological, evolutionary & environmental sciences

For a reference copy of the document with all sections, see nature.com/documents/nr-reporting-summary-flat.pdf

Life sciences study design

All studies must disclose on these points even when the disclosure is negative.

Sample size	This project was a retrospective study. No sample size calculation was performed. All patients with Parkinson's disease genetically characterized were included, and then selection criteria were applied. Serum total cholesterol, high-density and low-density lipoproteins and triglycerides were determined in peripheral blood and analyzed in the Central Laboratory at Hospital Universitario Virgen del Rocio, Seville, Spain.
Data exclusions	Data of patients with Parkinson's disease and data of healthy controls were excluded following the pre-established exclusion criteria. Also, data of those individuals with missing values were excluded too.
Replication	We provided a description of the entire methodology, for any other qualified researcher willing to replicate our study.
Randomization	No randomization was performed.
Blinding	Investigators were blinded during retrospective data collection (demographic and clinical data from both healthy controls and patients with Parkinson's disease) and analysis.

Reporting for specific materials, systems and methods

We require information from authors about some types of materials, experimental systems and methods used in many studies. Here, indicate whether each material, system or method listed is relevant to your study. If you are not sure if a list item applies to your research, read the appropriate section before selecting a response.

Materials & experimental systems

n/a	Involved in the study	
☒	☐	Antibodies
☒	☐	Eukaryotic cell lines
☒	☐	Palaeontology and archaeology
☒	☐	Animals and other organisms
☐	☒	Human research participants
☒	☐	Clinical data
☒	☐	Dual use research of concern

Methods

n/a	Involved in the study	
☒	☐	ChIP-seq
☒	☐	Flow cytometry
☒	☐	MRI-based neuroimaging

Human research participants

Policy information about studies involving human research participants

Population characteristics	This project was a retrospective study, including patients with Parkinson's disease (PD) from the Movement Disorder Clinic at Hospital Universitario Virgen del Rocio in Seville, Spain. PD was diagnosed following the Movement Disorder Society Clinical Diagnostic Criteria (Postuma et al. 2015). Patients with PD were classified into three subgroups: sporadic Parkinson's disease (sPD), GBA-associated Parkinson's disease (GBA-PD), and LRRK2-associated Parkinson's disease (LRRK2-PD). A control group with healthy controls was also included. All subjects considered for the study were Caucasian to avoid ethnic influences in the lipid profile. Patients with sPD were not considered for the study if they had any variants in other PD-related genes. LRRK2-PD group contained 28 LRRK2 p.G2019S PD patients (90.3 %) and 3 LRRK2 p.R1441G PD patients (9.7%). The list of GBA pathogenic variants considered for the inclusion of patients in the GBA-PD group is shown within the manuscript (Supplementary material 2). Exclusion criteria for all individuals included were receiving treatment with lipid-modifying therapy (i.e. statins, ezetimibe, antivirals, azathioprine); and having a first-degree PD family history exclusively for patients with sPD.
Recruitment	All subjects considered for the study were Caucasian to avoid ethnic influences in the lipid profile. Patients with PD were recruited from the Movement Disorder Clinic at Hospital Universitario Virgen del Rocio (Seville, Spain). Healthy controls were recruited from the same geographical area and they were not considered in the study if they had any neurodegenerative disorder, a family history of PD, or a variant in GBA or LRRK2 genes.
Ethics oversight	We obtained consent from the local ethics committee of our hospital (Hospitales Universitarios Virgen del Rocío y Virgen

Ethics oversight	Macarena, Seville, Spain) in accordance with the Declaration of Helsinki, and written informed consent from all the participants in the study.

Note that full information on the approval of the study protocol must also be provided in the manuscript.
